# Maternal Mental Health After Stillbirth: Associations With Autopsy Decisions, Cause of Death Knowledge, and Psychosocial Risk Factors

**DOI:** 10.1007/s10995-026-04314-8

**Published:** 2026-08-19

**Authors:** Rana Jawish, Emily DiBlasi, Amanda A. Allshouse, Renee Martin-Willett, Becky Kinkead, Matthew Moench, Paul J. Carlson, Marcela C. Smid, Adam J. Gordon, Robert M. Silver, Mark H. Rapaport

**Affiliations:** 1 Department of Psychiatry, Huntsman Mental Health Institute, University of Utah, 501 Chipeta Way, Salt Lake City, UT 84108 USA; 2 Department of Obstetrics and Gynecology, University of Utah, Salt Lake City, UT USA; 3 Department of Psychology & Neuroscience, University of Colorado Boulder, Boulder, CO USA; 4 Decision-Enhancement, and Analytic Sciences (IDEAS) Center, VA Salt Lake City Health Care System, Salt Lake City, UT USA; 5 Program for Addiction Research, Clinical Care, Knowledge and Advocacy (PARCKA), Division of Epidemiology, Department of Internal Medicine, University of Utah School of Medicine, Salt Lake City, UT USA; 6 American Psychiatry Association (APA), Washington, USA

**Keywords:** Stillbirth, Maternal mental health, Autopsy, Childhood trauma, Socioeconomic factors

## Abstract

**Purpose:**

Stillbirth is a traumatic event with potentially lifelong effects on maternal mental health, but there are few clinical guidelines or resources for supporting families. Additionally, the effects of parental autopsy decision-making and knowledge of cause of death effect on maternal mental health is unclear.

**Methods:**

We evaluated associations between autopsy decision, cause of death determination, and maternal mental health in data from the multi-center, five region Stillbirth Collaborative Research Network (2006-2008). Secondarily, we examined psychosocial risk factors associated with adverse MMH outcomes. Maternal mental health was assessed at single time point within 6–36 months after stillbirth delivery. Exposures included autopsy decision (none, partial, full) and knowledge of cause of death (known, unknown). Adverse maternal mental health was defined as abnormal scores on one or more validated instruments assessing depression, grief, distress, post-traumatic growth, and change in substance use. Psychosocial risk factors included childhood trauma and perceptions of support or blame. Logistic regression identified associations with maternal mental health outcomes.

**Results:**

Among 272 participants (44% response rate), 54% reported one adverse maternal mental health outcome 6–36 months postpartum. Autopsy decision and knowledge of cause of death did not impact maternal mental health. Knowledge of cause of death was associated with less feeling of blame (OR 2.02, 95% CI 1.05–3.88). Childhood trauma (OR 4.10, 95% CI 1.60–10.70) and low income (OR 2.32, 95% CI 1.34–4.02) were associated with poorer maternal mental health.

**Conclusions:**

Despite the impact of stillbirth on maternal health, our understanding of factors impacting mental health is limited, and the clinical guidelines for providing psychological care are limited. In the current study, autopsy decision and knowledge of cause of death did not impact mental health outcomes 6-36 months postpartum, while childhood trauma, feeling blamed for the loss, and socioeconomic disadvantage were significantly associated with adverse mental health. This important, early data from the Stillbirth Collaborative Research Network may help inform screening for psychosocial risk factors and updating guidelines for providing trauma-informed support following stillbirth.

**Supplementary Information:**

The online version contains supplementary material available at https://doi.org/10.1007/s10995-026-04314-8.

## Significance

More than half of women experienced adverse mental health outcomes following stillbirth. Psychosocial risk factors, rather than autopsy decision-making or knowledge of the cause of death, identified women at greatest risk, highlighting the need for trauma-informed bereavement care.

**Supplementary Information:**

The online version contains supplementary material available at https://doi.org/10.1007/s10995-026-04314-8.

## Introduction

Stillbirth (SB), defined as fetal death at or after 20 weeks’ gestation, affects approximately 1 in 150–175 births in the United States, accounting for more than 20,000 cases annually (Gregory et al., 2022; Sullivan et al., 2025). Despite its frequency, the cause of SB remains unknown in 25–60% of cases (Heazell et al., 2016). The American College of Obstetricians and Gynecologists (ACOG) recommends fetal autopsy, gross and histologic examination of the placenta, umbilical cord, and membranes in all cases of SB (ACOG Practice Bulletin No 102, 2009), with comprehensive autopsy providing cause of death (COD) in 40–70% of stillbirths (Page et al., 2017; Pinar et al., 2011). When only some of these components are conducted, the process is referred to as a partial autopsy (Oliver et al., 2022).

The impact of autopsy decision-making and knowledge of COD on maternal mental health (MMH) remains poorly understood. ACOG cites the value of SB evaluation in identifying modifiable risk factors and offering closure to families (American College of Obstetricians and Gynecologists, 2020), but families often experience emotional distress when deciding whether to pursue an autopsy, and little is known regarding the effect of this decision and subsequent knowledge of COD has on short and long term MMH outcomes (Schirmann et al., 2018). Women who experience SB have a twofold increased risk of post-loss depression and anxiety compared to those with live births (Herbert et al., 2022), along with a markedly elevated risk of maternal suicide in the first postpartum year (Weng et al., 2018). These psychological effects may persist for years, with studies consistently demonstrating elevated levels of distress well beyond the perinatal period (Hogue et al., 2015; Westby et al., 2021). Despite these risks, the 2024 Society for Maternal-Fetal Medicine President’s Workshop on Maternal Mental Health, explicitly acknowledged the lack of structured care pathways in SB, and highlighted ongoing stigma and misconceptions affecting bereaved parents, without indication that stillbirth-specific guidelines are forthcoming (Society for Maternal-Fetal Medicine. Electronic address, 2024). Current ACOG offers limited, non-specific recommendations for mental health care following stillbirth: advising referral to bereavement counseling or mental health professionals “when appropriate” without guidance on risk stratification or longitudinal follow-up, (ACOG Practice Bulletin No 102, 2009). Similarly, the American College of Obstetricians and Gynecologists (ACOG) Committee Opinion, Optimizing Postpartum Care, emphasizes emotional support and linkage to support groups but does not address the long-term psychiatric risks unique to stillbirth.(McKinney et al., 2018).

To address these gaps, this study examined associations between autopsy decisions, COD knowledge, and MMH outcomes 6 to 36 months after SB as well as explored psychosocial and medical factors associated with persistent distress. These findings may inform evidence-based, trauma-informed guidance to better identify and support women who experience SB and are at highest risk for adverse MMH outcomes.

## Materials and Methods

### Study Design and Population

The Stillbirth Collaborative Research Network (SCRN) was established in 2003 to investigate SBs in the U.S. through a multicenter, case-control study across five diverse regions. From 2006 to 2008, the study enrolled patients at the time of hospitalization for SB delivery at 59 hospitals, utilizing standardized protocols for data collection, maternal interviews, biospecimen analysis, placental pathology, and postmortem examinations. This resource has been previously described in detail (Parker et al., 2011). The SCRN-Outcomes after Study Index SB (OASIS) study followed up with SCRN participants at a single time point within 6 months to 3 years post-delivery. Participants were asked about subsequent pregnancies, complications, and life-course stresses that might be associated with an increased risk of SB as described previously (Hogue et al., 2015). Our study is a secondary analysis of the OASIS follow-up to the SCRN. We limited our analytical population to cases of singleton SB who participated in the follow-up questionnaire administered 6–36 months postpartum.

### Measures

Our primary exposure was a combination of autopsy decision (none, partial, or full autopsy) and COD (identified or not identified), defined as knowledge gained from autopsy (unexplored, unexplained, or known COD). Known COD was defined as COD identified following partial or full autopsy. Unexplored COD was defined as COD not identified following partial or no autopsy. Unexplained COD was defined as COD not identified following full autopsy.

COD was determined using the Initial Causes of Fetal Death (INCODE) algorithm that integrates clinical, placental, and autopsy findings to assign a “probable” or “possible” cause within standardized categories. Known COD was defined as identification of a probable or possible cause in any of eight INCODE categories: placental disease, infection, fetal genetic or structural abnormality, maternal medical complication, hypertensive disorder, cord abnormality, obstetric complication, or other specified condition (Stillbirth Collaborative Research Network Writing, 2011). Within the SCRN classification system, undetermined and unassigned COD categories represent cases in which a specific probable or possible cause of death could not be identified. Because both categories lack a definitive COD determination, they were combined as unknown COD for analytic purposes. Classification of COD required a full or partial autopsy. No COD was assigned to participants who declined autopsy.

The primary outcome was adverse MMH at follow-up, defined as an elevated score on one or more of five validated instruments assessing depression, grief, distress, post-traumatic growth (defined below for each scale specifically), and change in substance use at a single time point between 6 and 36 month post-delivery. Depression was assessed with the 10-item Edinburgh Depression Scale (EPDS); a score ≥ 11 indicated a positive depression screen (Cox et al., 1987; Levis et al., 2020; Pitanupong et al., 2007). Grief was included as an indicator of bereavement intensity and assessed using the 33-item Perinatal Grief Scale (Toedter et al., 1988). This measure is comprised of three subscales (active grief, difficulty coping, and despair) with scores ranging from 11 to 44 (higher scores indicate more intense grief). A sensitivity analysis excluding grief from the composite was performed to assess robustness, and we applied a threshold of > 33 as elevated in the absence of a universally established cutoff, consistent with the approach used by Lasker & Toedter in their scale validation. Subjective distress was measured using the 15-item Impact of Events Scale (IES) with higher scores indicating greater distress and a score > 44 indicating severe distress (Horowitz et al., 1979). Post-traumatic growth was measured using the 21-item Posttraumatic Growth Inventory (PTGI) scale (Tedeschi & Calhoun, 1996). The PTGI measures positive psychological changes following trauma, with higher scores denoting more growth and better adaptation following trauma. In the absence of a clinically accepted cutoff, we defined low growth as a PTGI score < 42, representing an average response of (2) “to a small degree” on each of the 21 items. Score calculation allowed for up to 4 missing items. Finally, self-reported increase (indicated by an affirmative response) in alcohol drinking or smoking habits, was assessed with the question “In those first two months, did your drinking habits change? Would you say you drank more or started drinking, or drank less or stopped drinking?” Similar language was used for smoking.

Demographic, clinical, psychological, medical, and systemic factors were evaluated as secondary risk or protective factors for MMH outcomes following SB. Demographic factors included time since index pregnancy, socioeconomic status/insurance as a proxy, age, social changes affecting job or relationship status, and other children (living children from previous pregnancies, current pregnancy status, live births since the index pregnancy, and adoption since the index pregnancy). Education, employment status, marital status, and living with a partner were collected at time of SB.

Clinical and psychological factors were defined as a history of mental health diagnosis or treatment (medication or psychotherapy) documented at SB delivery, or elevated symptom scores on questionnaires administered at the time of stillbirth. At the time of SB delivery, participants completed the 20-item Spielberger Trait Anxiety Scale of the State-Trait Anxiety Inventory (STAI), with a score ≥ 40 defined as high anxiety (Quek et al., 2004). Additionally, anger was evaluated at the time of SB delivery utilizing the Spielberger Trait Anger Scale of the State-Trait Anger Expression Inventory (STAXI-2) which consists of 57 items each rated on a 4-point scale (Lievaart et al., 2016). STAXI-2 is used to measure anger frequency, intensity, anger expression, and control. Depression history was assessed by asking “At any time in your life before this pregnancy, did you ever experience depression or feel “down in the dumps?” with possible response options ‘yes’ and ‘no’. At the time of follow-up interview (conducted 6–36 months after stillbirth), perceived blame was examined as an exploratory indicator of the social context surrounding stillbirth rather than as a diagnostic construct. Participants were asked, ‘Do you feel that someone blames you for the loss?’ This item was intended to capture external or enacted stigma from others, which differs from self-blame or guilt measured in prior studies. At the same follow-up interview, participants also completed the 28 item Childhood Trauma Questionnaire (CTQ) (Bernstein et al., 2003) which retrospectively assesses childhood maltreatment. A CTQ cutoff of > 35 was used to indicate a significant history of childhood trauma.

Medical comorbidities included specific chronic conditions (diabetes, coronary artery disease, hypertension, valvular and other structural heart disorders, kidney, sickle cell disease) and autoimmune conditions (systemic lupus erythematosus, rheumatoid arthritis, antiphospholipid syndrome, ulcerative colitis, Crohn’s disease), as well as blood clotting disorders or stroke. Pregnancy-related morbidity and exposures included gestational diabetes, gestational hypertension, cholestasis, preterm birth (< 37weeks), cesarean mode of delivery, and timing of stillbirth (intrapartum vs. antepartum). Participants were asked about types of support they received in the hospital at the time of delivery and through the first two months after SB; for each type of support there were two responses (yes and no). Participants were asked about support from doctors, MH professionals, the father of the baby, partners, family members, spiritual/religious support, hospital staff, and friends.

Demographic characteristics at the time of stillbirth delivery were compared between participants who completed the OASIS follow-up interview (included in the present analysis) and eligible women who did not complete the follow-up interview (non-participants) (Supplementary Table 1). Demographic characteristics were also compared by autopsy decision and COD knowledge exposure. MHH outcomes were similarly compared by autopsy decision and COD knowledge exposure.

Secondary analyses examined potential risk and protective factors associated with MMH outcomes. Bivariate comparisons used chi-square tests for categorical variables and t-tests (binary exposure) or ANOVA (three-category combined autopsy/COD exposure) for continuous variables. Factors by which autopsy choice and COD knowledge combined exposure groups differed, and factors associated with MMH outcome were considered for multivariable modeling; variables measuring similar underlying features were combined or screened prior to modeling. Variable selection was then performed using backwards selection, with variable removal when *p* > 0.05. Two multivariable logistic regression models were then developed. The primary model included pre-existing maternal characteristics and baseline clinical factors, whereas the secondary, exploratory model evaluated post-stillbirth psychosocial factors to examine their associations with maternal mental health outcomes. Data analysis was generated using SAS software, Version 9.4 of the SAS System for Windows (SAS Institute Inc. Cary, NC). Graphics were created using GraphPad Prism version 10.0.3 for Windows (GraphPad Software, La Jolla California). Sample size estimate was completed with PASS 2022 version 22.0.3, NCSS, LLC (Kaysville, Utah, USA, ncss.com/software/pass).

## Results

**Fig. 1:**
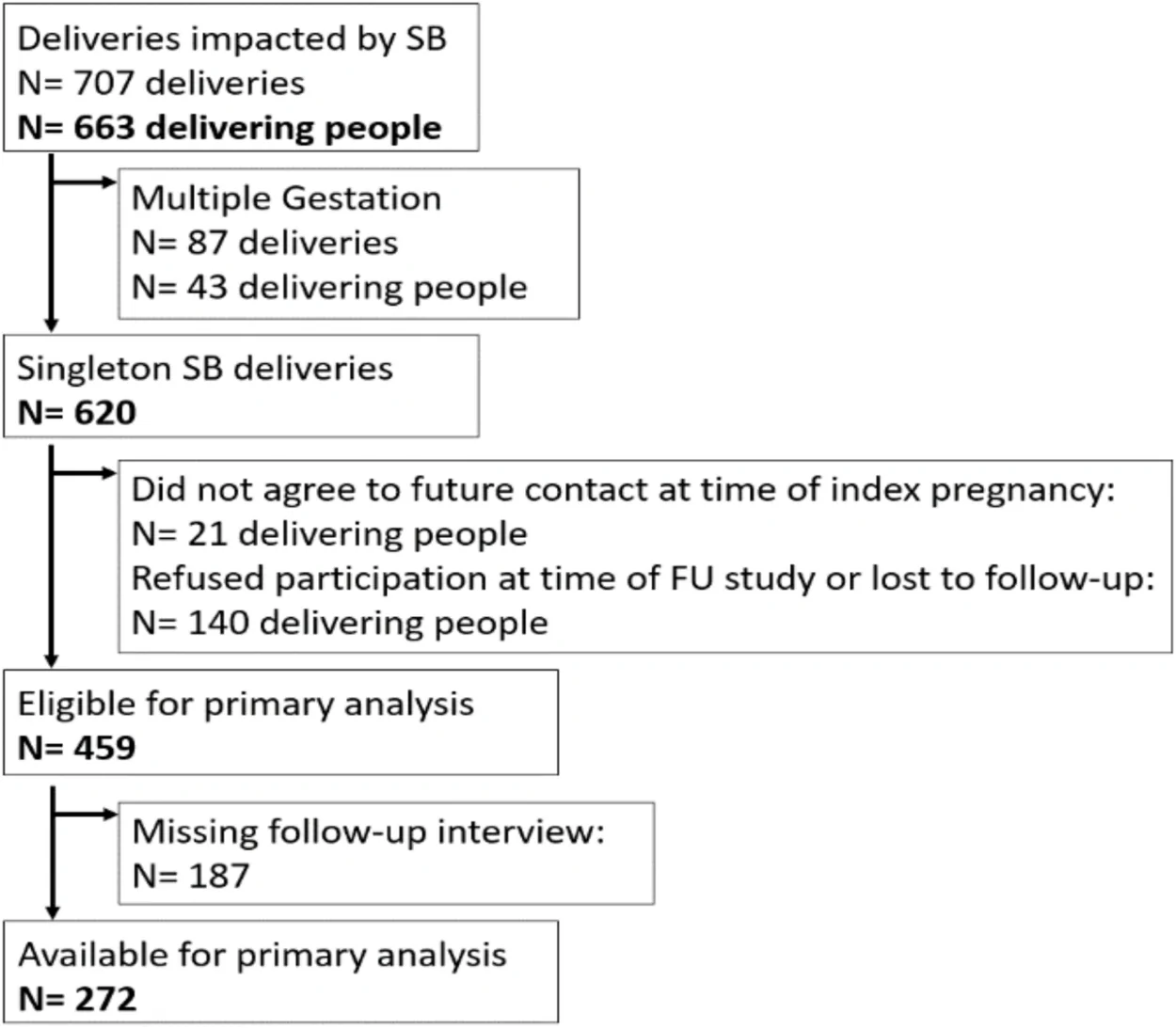
Flow diagram of participant selection. The diagram illustrates the derivation of the analytic cohort from the Stillbirth Collaborative Research Network (SCRN). Of 620 singleton stillbirth deliveries, 459 participants were eligible for OASIS follow-up, and 272 completed the follow-up interview and were included in this secondary analysis

Of the 620 women experiencing singleton SB deliveries, 272 (43.9%) completed follow-up interviews 10 to 39 months postpartum (Fig. 1). Inclusion in the present analysis was associated with higher maternal age, non-Hispanic ethnicity, commercial insurance, higher education, and being married (Supplementary Table 1). Full autopsy was selected by *n* = 181 (66.5%) participants, *n* = 52 (19.1%) selected partial autopsy, and *n* = 39 (14.3%) had no autopsy. COD was identified in 71.8% of cases with full autopsy, 51.9% with partial autopsy, and 0% of cases without autopsy (*p* < 0.001). When autopsy decision and COD knowledge were evaluated as a combined exposure, *n* = 157 (57.7%) had known COD, *n* = 51 (18.8%) had unexplained COD, and *n* = 64 (23.5%) had unexplored COD. Follow-up meantime after SB was 24.5 ± 7.7 months, with 48.2% of follow-up assessments occurring 2–3 years post SB (Table 1). Having experienced a subsequent pregnancy that had ended by the time of follow-up was more common among participants with unexplored COD (68.8%) and unexplained COD (58.8%) compared to those with a known COD (47.1%), whereas current pregnancy at follow-up was more frequent among participants with a known COD (18.4%) than among those with unexplained (12.2%) or unexplored COD (3.3%) (Table 1).

Poor MMH at follow-up (an abnormal score on one or more of the five validated instruments) was present for 54.4% (95%CI: 48.5–60.4%) of the cohort, 54.1% of participants with known COD, 58.8% for participants with unexplained COD, and 51.6% for participants with unexplored COD (Table 2). When the mental-health composite was evaluated over time after stillbirth by quarter year increment, there was not an apparent directional trend over time (chi-square test for trend *p* = 0.35). Exposure categories did not differ between women with and without poor MMH outcomes, and individual MMH instrument components similarly showed no differences by exposure. MMH outcomes also did not vary by autopsy decision or identified COD when examined individually (Online Appendix Table 2).

Of the 148 participants with poor MMH at follow-up, the majority had poor outcomes on only 1 (29.0%) or 2 (14.7%) of the evaluated mental health outcomes (Table 2). 26% scored high for depression, 25% had elevated distress, and 18% were experiencing grief. 15% reported increased smoking or drinking in the two months after the SB, and 11% had low resilience (Table 2). Poor MMH outcomes were significantly associated with higher scores for childhood physical abuse (specifically emotional abuse, emotional neglect, and physical neglect), lower minimization/denial scores, a history of mental health conditions during pregnancy, autoimmune disorders, feeling blamed by the fetus’ father, less support from friends and mental health counselors in the first two months following stillbirth, lower household income, and greater anger expression at the time of stillbirth (Table 3, 4, 5).

Most participants were counseled about holding or seeing their infant (93.8%) and received mementos or keepsakes (92.6%) (Table 5). Most participants also reported support from partners and family members. However, those with adverse psychosocial outcomes were significantly less likely to report support from friends or from a counselor or mental-health professional (Table 5). Engagement in these practices was not significantly associated with poorer mental-health outcomes.

In multivariable analyses, the combined exposure comparing women with known COD, unexplained COD, and unexplored COD was not significantly associated with poor MMH and was removed for parsimony. The primary multivariable model included pre-existing maternal characteristics and baseline clinical factors and included only variables known at or before the time of SB or earlier. Autoimmune diagnosis was removed during variable selection because no adverse MMH outcomes occurred among women with autoimmune disease. Among remaining candidates, two remained after variable selection: severe emotional or physical abuse or neglect in childhood (OR 4.14, 95% CI: 1.61–10.7) and receiving income assistance (vs. only personal income) (OR 2.32, 95% CI: 1.34–4.02). In the secondary exploratory model, post-stillbirth psychosocial factors (perceived blame and support from friends and mental health professionals during the first two months after stillbirth) were additionally evaluated. Support from a mental health professional remained associated with poor MMH (OR 2.98, 95% CI: 1.01–8.85), while the associations with childhood trauma and income assistance remained similar in magnitude and statistically significant.

When grief was excluded from the composite, results were generally unchanged. Associations with income assistance (OR 2.32, 95% CI 1.35–3.99) and childhood trauma (OR 4.17, 95% CI 1.70–10.25) remained significant and similar in magnitude, whereas the association with therapist support was attenuated (OR 2.12, 95% CI 0.77–5.83).

## Discussion

These secondary analyses of a large multisite follow up cohort remain among the few U.S. studies to date to examine associations between maternal mental health, cause of death determination, autopsy findings, and individual risk factors following stillbirth. Despite growing recognition of the substantial psychiatric burden after stillbirth, including elevated risks of depression, anxiety, PTSD, substance use, and suicide, professional guidelines in the U.S. continue to lack evidence-based protocols addressing these outcomes beyond general bereavement support. Thus, although data collection was completed in 2008, the findings remain highly relevant given this persistent absence of standardized, evidence-based mental health care guidelines specific to women who experience stillbirth.

Among 272 participants assessed 6–36 months after stillbirth, more than half reported at least one adverse MMH outcome, underscoring the substantial and persistent psychiatric burden following loss. While neither undergoing autopsy nor having an autopsy-identified COD was significantly associated with MMH outcomes 6–36 in this sample, participants with a known COD reported lower levels of perceived blame from others. This finding suggests that diagnostic clarification may influence the social context surrounding stillbirth, even if it does not translate into measurable improvements in long-term mental health. Given the strong association between perceived blame and adverse MMH outcomes in this cohort, addressing blame and stigma may represent an important target for post-stillbirth care.

Several other factors were significantly associated with poor MMH outcomes, including a history of childhood trauma, low income or receipt of public assistance, perceived blame from others (particularly from the child’s father), limited social support, and autoimmune diagnoses. These findings underscore the complex, multifactorial nature of psychological distress after SB and highlight the need for trauma-informed interventions (Westby et al., 2021). Our study aligns with previous work demonstrating self-blame and emotional avoidance following SB were strong predictors of depression following loss (Balle et al., 2024), while guilt and stigma have also been identified as key contributors to PTSD and depressive symptoms in this context (Burden et al., 2016). Importantly, these pathways are distinct from grief, which is a normative response to loss. However, its intensity can influence functioning and coping (Burden et al., 2016), and while excluding grief in sensitivity analyses did not alter the main findings, the attenuation of the therapist-support association suggests grief may contribute uniquely to psychosocial experience after stillbirth. Future research should test whether psychosocial counseling, particularly during autopsy result disclosure, can ease guilt, reduce blame, and improve maternal mental health outcomes.

Significant associations between MMH, severe childhood emotional or physical abuse or neglect, and receipt of public income assistance align with broader perinatal mental-health research showing that pre-existing vulnerabilities such as adverse childhood experiences and socioeconomic hardship are strong predictors of postpartum depression after live birth (Cooke et al., 2025; Fu et al., 2024). These associations also support the notion that screening for trauma and social stressors can be particularly fruitful in identifying risk for adverse MMH, given a history of childhood trauma often predicts poorer adult mental health and weakened social networks in general (Schneider et al., 2020). Few studies however have directly examined how preconception trauma shapes MMH after stillbirth, a critical gap in the bereavement literature. Encouragingly, supportive relationships emerged as a protective factor in our analyses. Participants with poor MMH outcomes were less likely to report receiving emotional support from friends and professional counselors in the immediate post-loss period, suggesting the development of proactive outreach systems to reduce isolation, increase access to care, and facilitate healthy coping.

Several limitations of our study should be acknowledged. These data were collected over 15 years ago, prior to the widespread adoption of structured bereavement care protocols and expanded peer support networks. Since that time, access to psychosocial and trauma informed care has improved, and contemporary experiences may differ from those captured in this cohort. However, the 2023 ACOG Clinical Practice Guideline on perinatal mental health still does not identify stillbirth as requiring distinct screening or follow-up strategies despite these advances in the field (ACOG, 2023), and these data remain among the few population-based, systematic studies linking medical causes of stillbirth with validated measures of maternal mental health, offering an important historical benchmark for understanding progress in perinatal bereavement care.

Second, our modest sample size and participation rate may limit generalizability, and reliance on self-report introduces the potential for recall and social desirability bias. Roughly 44% of the initial sample participated in the follow-up interview and were more likely to be older, white, married, and commercially insured, suggesting individuals with fewer resources or greater distress may have been underrepresented. Additionally, while we used validated tools to assess symptoms of depression, distress, and grief, formal clinical diagnoses and assessments were not made, and for some outcome scales, we lacked evidence-based cutoffs to define poor outcomes and partner’s mental-health status was not assessed.

Finally, several psychosocial factors, including perceived blame and social support, were assessed concurrently with maternal mental health outcomes and timing of follow-up varied across participants between 10 and 39 months after stillbirth, thus precluding determination of temporality. Because maternal grief and psychological recovery evolve over time, this variability may have introduced heterogeneity in symptom severity, with the greatest decrement in well-being occurring during the first six months after loss, though the trajectory of recovery thereafter was inconsistent (Camacho et al., 2024). Of note, our sample did not include women within that earliest, most acute period; the first response occurred at month 10. While time since loss was adjusted for in all models and confidence intervals for the mental-health composite score overlapped across quarters with an overall prevalence estimate of 54% suggesting relative stability of results, findings should be interpreted as representing the 6 to 36-months post-loss period rather than the immediate bereavement phase.

## Conclusion

In this study, autopsy decisions and cause-of-death (COD) knowledge did not impact MMH outcomes 6–36 months postpartum, although knowing the COD may reduce maternal self-blame and facilitate grief processing. In contrast, childhood trauma, perceived blame, and socioeconomic disadvantage were strongly associated with adverse mental health outcomes. These findings underscore the importance of psychosocial risk screening and trauma-informed, longitudinal support to optimize maternal mental health following stillbirth.

**Table 1:** Demographic characteristics of participants based on the combined impact of the decision to autopsy and knowledge of the cause of death (COD)

Characteristic	Value	Unexplored COD *N* = 64	Unexplained COD *N* = 51	Known COD *N* = 157	*p*-value
Time since stillbirth delivery	Mean ± SD (months)	25.1 ± 7.9	24.2 ± 7.9	23.8 ± 7.8	0.536
	6–23 months	25 (39.1)	20 (39.2)	73 (46.8)	0.092
	24–35 months	29 (45.3)	29 (56.9)	73 (46.8)	
	36–42 months	10 (15.6)	2 (3.9)	10 (6.4)	
Maternal age in years at delivery	Mean ± SD	27.0 ± 6.4	27.0 ± 6.0	28.4 ± 6.7	0.229
	< 20	8 (12.5)	4 (7.8)	15 (9.6)	0.318
	20–34	45 (70.3)	42 (82.4)	111 (70.7)	
	35–39	10 (15.6)	2 (3.9)	24 (15.3)	
	40+	1 (1.6)	3 (5.9)	7 (4.5)	
Maternal BMI at delivery	Mean ± SD (Kg/m^2^)	27.8 ± 7.5	27.9 ± 7.2	27.8 ± 6.9	0.994
	< 18.5 (underweight)	1 (1.7)	4 (8.0)	4 (2.6)	
	18.5–24.9 (normal weight)	25 (42.4)	14 (28.0)	60 (39.0)	
	25–29.9 (overweight)	15 (25.4)	16 (32.0)	39 (25.3)	
	30–34 (obese)	10 (16.9)	8 (16.0)	25 (16.2)	
	35+ (morbidly obese)	8 (13.6)	8 (16.0)	26 (16.9)	
Maternal race/ethnicity	white non-Hispanic	35 (54.7)	28 (54.9)	68 (43.3)	0.551
	black, non-Hispanic	10 (15.6)	7 (13.7)	30 (19.1)	
	Hispanic	18 (28.1)	13 (25.5)	50 (31.8)	
	other	1 (1.6)	3 (5.9)	9 (5.7)	
Insurance/method of payment at delivery	Government assistance or not insured	29 (45.3)	19 (37.3)	77 (49.4)	0.318
Family income source in 12 months before delivery	only public/private assistance	1 (1.8)	1 (2.0)	8 (5.4)	0.722
	assistance and personal income	18 (32.7)	16 (32.7)	43 (29.1)	
	only personal income	36 (65.5)	32 (65.3)	97 (65.5)	
Maternal education at delivery	Mean ± SD (years)	13.7 ± 2.4	14.1 ± 2.5	13.5 ± 2.7	0.365
	Less than college	24 (42.9)	19 (38.0)	69 (46.0)	0.622
	College or more	32 (57.1)	31 (62.0)	81 (54.0)	
Marital status/cohabitating at delivery	Not married/cohabitating	6 (10.7)	6 (12.0)	32 (21.3)	0.114
	Cohabitating/married	50 (89.3)	44 (88.0)	118 (78.7)	
Obstetric history at time of delivery	Nulliparous	33 (52.4)	27 (52.9)	75 (47.8)	0.736
	First pregnancy	18 (28.1)	16 (31.4)	50 (31.8)	0.873
	Previous Stillbirth	3 (6.8)	1 (2.9)	9 (8.4)	0.623
	Pregnancy wanted	52 (92.9)	48 (98.0)	140 (94.0)	0.552
	Assisted reproductive technology	5 (7.8)	3 (5.9)	10 (6.4)	0.892
Since delivery	**Completed pregnancy**	**44 (68.8)**	**30 (58.8)**	**74 (47.1)**	**0.011**
	**Currently Pregnant**	**2 (3.3)**	**6 (12.2)**	**28 (18.4)**	**0.014**
	Adopted Children	1 (1.6)	2 (3.9)	4 (2.5)	0.755

*COD* Cause of death, *SB* Stillbirth, *BMI* Body mass index, *HMO* Health maintenance organization, *SD* Standard deviation. Bolded values indicate statistical significance. Values are expressed as N (column %) or Mean±Standard Deviation.

**Table 2:** Maternal Mental Health Outcomes and the Impact of Combined Autopsy Decision and Cause of Death (COD) 6–36 Months Post Stillbirth Delivery

Outcome		Combined Exposure			
	Whole Cohort *N* = 272	Unexplored COD *N* = 64	Unexplained COD *N* = 51	Known COD *N* = 157	Combined Exposure *P* value
Number of ‘Poor’ maternal mental health outcomes					
0	124 (45.6)	31 (48.4)	21 (41.2)	72 (45.9)	0.855
1 or more	148 (54.4)	33 (51.6)	30 (58.8)	85 (54.1)	0.746
1	79 (29.0)	18 (28.1)	17 (33.3)	44 (28.0)	
2	40 (14.7)	7 (10.9)	7 (13.7)	26 (16.6)	
3	18 (6.6)	5 (7.8)	4 (7.8)	9 (5.7)	
4	10 (3.7)	2 (3.1)	2 (3.9)	6 (3.8)	
5	1 (0.4)	1 (1.6)	0 (0.0)	0 (0.0)	
Number of participants scoring ‘Poor’ on individual scales (N (%))					
Edinburgh Depression Scale (EPDS) ≥ 11	72 (26.6)	21 (33.3)	14 (27.5)	37 (23.6)	0.342
Perinatal Grief Scale - Any subscale > 33	51 (18.8)	8 (12.5)	10 (19.6)	33 (21.0)	0.319
Active Grief subscale >33	49 (18.1)	8 (12.7)	9 (17.6)	32 (20.4)	0.412
Difficulty Coping subscale >33	49 (18.1)	8 (12.7)	9 (17.6)	32 (20.4)	0.412
Despair subscale > 33	4 (1.5)	0 (0.0)	1 (2.0)	3 (1.9)	0.64
Impact of Events Scale (IES) > 44	67 (24.7)	17 (27.0)	11 (21.6)	39 (24.8)	0.791
Posttraumatic Growth Inventory (PTGI) scale < 42	29 (10.7)	4 (6.3)	6 (11.8)	19 (12.1)	0.452
Increased smoking/drinking	39 (14.8)	10 (16.1)	10 (20.0)	19 (12.5)	0.399
Individual Scale and subscale scores (mean ± SD)					
Edinburgh Depression Scale (EPDS)	7.4 ± 5.2	8.2 ± 5.6	7.2 ± 5.4	7.1 ± 5.0	0.353
Perinatal Grief Scale - Active Grief subscale	27.3 ± 6.3	26.6 ± 6.0	27.0 ± 5.8	27.6 ± 6.6	0.540
Perinatal Grief Scale - Difficulty Coping subscale	19.6 ± 6.2	19.5 ± 5.4	19.6 ± 6.3	19.6 ± 6.5	0.986
Perinatal Grief Scale - Despair subscale	19.1 ± 6.0	19.0 ± 5.7	18.9 ± 6.4	19.2 ± 6.1	0.956
Impact of Events Scale (IES)	31.1 ± 18.1	33.5 ± 16.7	28.2 ± 19.4	31.0 ± 18.3	0.297
Posttraumatic Growth Inventory Scale (PTGI)	70.3 ± 21.4	72.4 ± 19.9	72.1 ± 22.5	68.8 ± 21.7	0.421

Edinburgh Depression Scale (EPDS), a score ≥ 11 indicated a positive depression screen; Grief was assessed using the 33-item Perinatal Grief Scale(Toedter et al., 1988), which comprises three subscales; Active Grief, Difficulty Coping, and Despair. The Perinatal Grief Scale yields scores ranging from 33 to 165 (higher scores indicate more intense grief). Subjective Distress was measured using the 15-item Impact of Events Scale (IES); higher scores indicate greater distress, and a score > 24 indicates severe distress (Horowitz et al., 1979). Post-traumatic growth was measured using the 21-item Posttraumatic Growth Inventory (PTGI) scale (Tedeschi & Calhoun, 1996). The PTGI measures positive psychological changes following trauma. Higher scores denote more growth and better adaptation following trauma. In the absence of a clinically meaningful cutoff for the PGTI, we defined low growth as a PGTI score < 42, representing an average response of (2) “to a small degree” on each of the 21 items. Values expressed as N (column %) or Mean±Standard Deviation. Categorical variables were compared using chi-square tests and continuous variables using one-way analysis of variance (ANOVA).

**Table 3:** Demographics and obstetric history at SB delivery by Maternal Mental Health (MMH) outcome

Factor	Value	Poor MMH	Otherwise	*p*
		*N* = 148	*N* = 124	
Time since SB for follow-up (months)	Mean ± SD	23.8 ± 8.0	24.7 ± 7.6	0.332
	6–23 months	66 (44.9)	52 (41.9)	0.848
	24 to less than 36 months	70 (47.6)	61 (49.2)	
	36 months to 42 months	11 (7.5)	11 (8.9)	
Maternal age (years) at delivery	Mean ± SD	27.8 ± 6.8	27.8 ± 6.3	0.951
	< 20	16 (10.8)	11 (8.9)	0.848
	20–34	105 (70.9)	93 (75.0)	
	35–39	20 (13.5)	16 (12.9)	
	40+	7 (4.7)	4 (3.2)	
Maternal BMI (kg/m^2^) at delivery	Mean ± SD	28.5 ± 7.3	27.0 ± 6.7	0.073
	< 18.5 (underweight)	4 (2.8)	5 (4.1)	0.493
	18.5–24.9 (normal weight)	48 (33.8)	51 (42.1)	
	25–29.9 (overweight)	38 (26.8)	32 (26.4)	
	30–34 (obese)	27 (19.0)	16 (13.2)	
	35+ (morbidly obese)	25 (17.6)	17 (14.0)	
Maternal race/ethnicity	White, non-Hispanic	71 (48.0)	60 (48.4)	0.802
	Black, non-Hispanic	24 (16.2)	23 (18.5)	
	Hispanic	47 (31.8)	34 (27.4)	
	other	6 (4.1)	7 (5.6)	
Insurance/method of payment at delivery	Government assistance or not insured	74 (50.3)	51 (41.1)	0.13
Family income last 12 months	Only public/private assistance	**7 (5.1)**	**3 (2.6)**	**0.013**
	Assistance and personal income	51 (37.5)	26 (22.4)	
	Only personal income	78 (57.4)	87 (75.0)	
Maternal education (years) at delivery	Mean ± SD	13.4 ± 2.7	13.9 ± 2.5	0.173
	Less than college	68 (49.3)	44 (37.3)	0.054
	College or more	70 (50.7)	74 (62.7)	
Marital status/cohabitating at delivery	Not married/cohabitating	24 (17.4)	20 (16.9)	0.926
	married/cohabitating	114 (82.6)	98 (83.1)	
OB history at delivery	Nulliparous	72 (48.6)	63 (51.2)	0.673
	First pregnancy	45 (30.4)	39 (31.5)	0.852
	Previous SB	9 (8.8)	4 (4.8)	0.28
	Pregnancy wanted	126 (92.6)	114 (96.6)	0.167
	Assisted reproductive technology	9 (6.1)	9 (7.3)	0.697
Since Stillbirth Delivery	Completed pregnancy	74 (50.0)	74 (59.7)	0.11
	Currently Pregnant	16 (11.3)	20 (16.7)	0.206
	Adopted Children	3 (2.0)	4 (3.2)	0.54

Bolded values indicate statistical significance

**Table 4:** Impact of mental health history and medical history on maternal mental health outcomes 6 months to 3 years after stillbirth (SB)

Factor	Value	Poor MH	Otherwise	*p*
		*N* = 148	*N* = 124	
History of Mental Health Condition	Mental health diagnosis prior to SB delivery	21 (14.2)	12 (9.7)	0.256
	Mental health diagnosis prior to SB pregnancy	21 (15.2)	11 (9.3)	0.155
	Mental health diagnosis during SB pregnancy	**14 (10.1)**	**3 (2.5)**	**0.015**
	Mental health meds during SB pregnant	**10 (7.2)**	**1 (0.8)**	**0.012**
Anxiety at time of delivery	High (STAI > = 40)	**61 (46.2)**	**33 (28.4)**	**0.004**
Trait anger scale (Staxi-2) total score	Mean ± SD	16.9 ± 4.9	16.9 ± 5.7	0.936
• Angry temperament subscale	Mean ± SD	6.1 ± 2.3	6.3 ± 2.6	0.532
• Angry reaction subscale	Mean ± SD	7.9 ± 2.8	7.5 ± 2.7	0.276
• Anger expression-out subscale	Mean ± SD	15.7 ± 3.9	15.5 ± 4.2	0.799
• Anger expression-in subscale	Mean ± SD	**13.6 ± 3.9**	**12.4 ± 3.7**	**0.017**
• Anger control-out subscale	Mean ± SD	23.4 ± 4.8	24.1 ± 5.4	0.295
• Anger control-in subscale	Mean ± SD	22.8 ± 5.1	23.2 ± 5.1	0.573
• Anger expression index	Mean ± SD	31.0 ± 13.4	28.6 ± 14.6	0.191
CTQ Overall Score	High (CTQ > 35)	32 (21.6)	16 (12.9)	0.078
• Emotional abuse subscale	Mean ± SD	**8.8 ± 5.0**	**6.7 ± 2.4**	**< 0.001**
• Physical abuse subscale	Mean ± SD	**7.3 ± 4.0**	**6.1 ± 2.0**	**0.002**
• Sexual abuse subscale	Mean ± SD	7.4 ± 5.3	6.6 ± 4.2	0.163
• Emotional neglect subscale	Mean ± SD	**9.3 ± 4.7**	**7.3 ± 3.2**	**< 0.001**
• Physical neglect subscale	Mean ± SD	**7.1 ± 3.2**	**5.8 ± 1.9**	**< 0.001**
• Minimization/denial	Mean ± SD	**9.9 ± 3.2**	**11.6 ± 2.8**	**< 0.001**
Severe CTQ response in any domain	Yes	32 (21.6)	16 (12.9)	0.078
• Emotional Abuse subscale severe	Yes	**18 (12.2)**	**2 (1.6)**	**< 0.001**
• Physical Abuse subscale severe	Yes	**15 (10.1)**	**4 (3.2)**	**0.026**
• CTQ Sexual Abuse subscale severe	Yes	21 (14.2)	13 (10.5)	0.357
• CTQ Emotional Neglect subscale severe	Yes	**12 (8.1)**	**2 (1.6)**	**0.016**
• CTQ Physical Neglect subscale severe	Yes	**13 (8.8)**	**3 (2.4)**	**0.026**
Chronic medical condition	Yes	30 (20.3)	16 (12.9)	0.106
Autoimmune medical condition	Yes	**5 (3.4)**	**0 (0.0)**	**0.039**
Blood Clotting or Stroke	Yes	2 (1.4)	0 (0.0)	0.194
Gestational diabetes	Yes	6 (4.1)	2 (1.6)	0.235
Preeclampsia/hypertension	Yes	14 (10.0)	10 (8.4)	0.659
Condition: cholestasis: during pregnancy	Yes	0 (0.0)	1 (0.8)	0.279
Intrapartum stillbirth	Yes	27 (18.2)	21 (16.9)	0.778
Type of delivery	spontaneous vaginal	112 (75.7)	111 (89.5)	0.079
	vacuum	1 (0.7)	1 (0.8)	
	vacuum & forceps	1 (0.7)	0 (0.0)	
	cesarean after labor	11 (7.4)	5 (4.0)	
	cesarean – no labor	21 (14.2)	6 (4.8)	
	other	2 (1.4)	1 (0.8)	
Timing of SB	Preterm Birth (PTB)	124 (83.8)	103 (83.1)	0.874

Bolded values indicate statistical significance

**Table 5:** Impact of System factors on maternal mental health (MMH) outcomes 6 months to 3 years after stillbirth (SB) delivery

Factor	Value	Poor MMH	Otherwise	*p*
		*N* = 148	*N* = 124	
Level of autopsy agreed to:	None	19 (12.8)	20 (16.1)	0.72
	Limited	28 (18.9)	24 (19.4)	
	Full	101 (68.2)	80 (64.5)	
Known COD	Yes	85 (57.4)	72 (58.1)	0.916
Support After Delivery While Still in Hospital	Yes	133 (90.5)	113 (91.1)	0.853
Support After Delivery: Doctor	Yes	94 (71.2)	75 (67.0)	0.474
Support After Delivery: Hospital nursing staff	Yes	109 (82.6)	98 (87.5)	0.285
Support After Delivery: Hospital grief personnel	Yes	77 (61.6)	63 (57.8)	0.554
Support After Delivery: Pastor/religious counselor	Yes	71 (56.3)	61 (56.5)	0.984
Support After Delivery: Support group	Yes	23 (18.4)	19 (17.9)	0.926
Support After Delivery: Father of the baby	Yes	111 (83.5)	100 (88.5)	0.26
Support After Delivery: Partner (not father of the baby) at the time of loss	Yes	17 (23.6)	9 (17.3)	0.395
Support After Delivery: Family member	Yes	118 (88.7)	102 (90.3)	0.695
Support After Delivery: Friend(s)	Yes	**92 (69.2)**	**90 (80.4)**	**0.046**
Support After Delivery: Other	Yes	28 (21.2)	33 (29.7)	0.127
Hospital Staff Discussed Seeing/Holding Baby	Yes	136 (93.8)	112 (91.1)	0.396
Discussed Seeing/Holding Baby: Decision	Saw	35 (23.8)	21 (16.9)	0.287
	Held	100 (68.0)	95 (76.6)	
	Neither	12 (8.2)	8 (6.5)	
Memorialized Baby: Given Mementos	Yes	137 (92.6)	119 (96.0)	0.235
Memorialize Baby: Memorial Service	Yes	95 (64.6)	76 (61.3)	0.571
Blamed for Loss: By anyone	Yes	**30 (20.3)**	**14 (11.3)**	**0.045**
Blamed for Loss: Father of the Baby	Yes	**14 (46.7)**	**2 (14.3)**	**0.038**
Blamed for Loss: Family Member	Yes	9 (30.0)	6 (42.9)	0.402
Blamed for Loss: Friend(s)	Yes	1 (3.3)	2 (14.3)	0.179
Blamed for Loss: Others	Yes	17 (56.7)	7 (50.0)	0.679
First Two Months: Any Support	Yes	132 (90.4)	111 (91.0)	0.872
Support First Two Months: Doctor	Yes	48 (36.4)	52 (47.7)	0.075
Support First Two Months: Hospital nursing staff	Yes	47 (35.9)	37 (33.6)	0.716
Support First Two Months: Hospital grief personnel	Yes	44 (34.9)	36 (33.0)	0.760
Support First Two Months: Pastor/Religious Counselor	Yes	49 (38.9)	46 (42.2)	0.606
Support First Two Months: Support Group	Yes	34 (26.4)	22 (20.4)	0.280
Support First Two Months: Father of the baby	Yes	112 (84.8)	101 (91.0)	0.147
Support First Two Months: Partner at that time	Yes	26 (35.6)	12 (21.8)	0.091
Support First Two Months: Family Member	Yes	112 (84.8)	100 (90.1)	0.222
Support First Two Months: Friend(s)	Yes	**92 (69.7)**	**90 (81.1)**	**0.041**
Support First Two Months: Other	Yes	30 (22.9)	24 (22.2)	0.901
First Two Months: Professional MH Support	Yes	32 (21.8)	24 (19.7)	0.673
ANY support from MH professional (* below)	Yes	22 (14.0)	13 (11.3)	0.510
• Psychiatrist*	Yes	7 (21.9)	3 (12.5)	0.365
• Psychologist*	Yes	4 (12.5)	5 (20.8)	0.401
• Counselor*	Yes	**16 (50.0)**	**5 (20.8)**	**0.026**
• Social worker*	Yes	2 (6.3)	2 (8.3)	0.765
Professional Support: Obstetrician	Yes	14 (43.8)	11 (45.8)	0.877
Professional Support: Others	Yes	7 (21.9)	5 (21.7)	0.990
First Two Months: Medications	Yes	50 (34.0)	33 (27.0)	0.218
• Medications Reason: Help Sleep	Yes	29 (58.0)	16 (48.5)	0.394
• Medications Reason: Reduce Anxiety	Yes	14 (28.0)	10 (31.3)	0.752
• Medications Reason: Reduce Depression	Yes	20 (40.0)	14 (42.4)	0.826
• Medications Reason: Others	Yes	17 (34.0)	8 (24.2)	0.343

Bolded values indicate statistical significance

## Supplementary Information

Below is the link to the electronic supplementary material.

Supplementary Material 1

## Data Availability

The data used in this manuscript are available on request to the corresponding author.

## Code Availability

The code would be provided on request to the corresponding author.
