# Supplementary material for: Maternal Mental Health After Stillbirth: Associations With Autopsy Decisions, Cause of Death Knowledge, and Psychosocial Risk Factors
Source: Matern Child Health J. 2026 Aug 19;30(9):1191–204. doi: 10.1007/s10995-026-04314-8 (PMC13569550; doi:10.1007/s10995-026-04314-8)
Supplement: Supplementary file 1 [file 10995_2026_4314_MOESM1_ESM.docx]

**Appendix Table 1. Characteristics, obstetric, and medical history of patients followed in the Stillbirth Collaborative Research Network (SCRN) who were eligible, but did or did not participate, in the follow-up (SCRN-Outcomes after Study Index Stillbirth (OASIS)) study.**

| **Characteristic** | **Value** | **Participants Not included in secondary analysis** | **Participated included in the secondary analysis** | **p** |
| --- | --- | --- | --- | --- |
|  |  | N= 348 | N= 272 |  |
| Agreed to Full Autopsy |  | 235 (67.7) | 181 (66.5) | 0.756 |
| Known Cause of Death |  | 194 (55.7) | 157 (57.7) | 0.623 |
| Combined Exposure | Unexplored | 93 (26.7) | 64 (23.5) | 0.655 |
|  | Unexplained | 61 (17.5) | 51 (18.8) |  |
|  | known COD | 194 (55.7) | 157 (57.7) |  |
| Maternal age at delivery | Mean±SD | **26.8±6.8** | **27.8±6.5** | **0.048** |
|  | <20 | 54 (15.5) | 27 (9.9) | 0.206 |
|  | 20-34 | 242 (69.5) | 198 (72.8) |  |
|  | 35-39 | 38 (10.9) | 36 (13.2) |  |
|  | 40+ | 14 (4.0) | 11 (4.0) |  |
| Maternal BMI | Mean±SD | 27.7±7.6 | 27.8±7.0 | 0.843 |
|  | 18.5 (underweight) | 18 (5.5) | 9 (3.4) | 0.669 |
|  | 18.5-24.9 (normal weight) | 133 (40.3) | 99 (37.6) |  |
|  | 25.0-29.9 (overweight) | 82 (24.8) | 70 (26.6) |  |
|  | 30.0-34.9 (obese) | 46 (13.9) | 43 (16.3) |  |
|  | 35.0+ (morbidly obese) | 51 (15.5) | 42 (16.0) |  |
| Maternal race/ethnicity | White, non-Hispanic | **90 (25.9)** | **131 (48.2)** | **<.001** |
|  | Black, non-Hispanic | 87 (25.1) | 47 (17.3) |  |
|  | Hispanic | 141 (40.6) | 81 (29.8) |  |
|  | other | 29 (8.4) | 13 (4.8) |  |
| Insurance/method of payment | any assistance or no insurance | **238 (69.0)** | **125 (46.1)** | **<.001** |
|  | commercial/ HMO / Military | 107 (31.0) | 146 (53.9) |  |
| Family income last 12 months | only public/private assistance | **36 (11.4)** | **10 (4.0)** | **<.001** |
|  | assistance and personal income | 135 (42.9) | 77 (30.6) |  |
|  | only personal income | 144 (45.7) | 165 (65.5) |  |
| Maternal education, years | Mean±SD | **12.1±3.0** | **13.6±2.6** | **<.001** |
|  | Less than college | **195 (61.7)** | **112 (43.8)** | **<.001** |
|  | College or more | 121 (38.3) | 144 (56.3) |  |
| Marital status/cohabitating | Not married / cohabitating | **96 (30.2)** | **44 (17.2)** | **<.001** |
|  | Married / cohabitating | 222 (69.8) | 212 (82.8) |  |
| OB history at SCRN delivery | nulliparous | 149 (42.9) | 135 (49.8) | 0.089 |
|  | First pregnancy | 106 (30.5) | 84 (30.9) | 0.929 |
|  | Previous SB | 27 (11.2) | 13 (7.0) | 0.138 |
|  | Pregnancy wanted | 293 (92.7) | 240 (94.5) | 0.395 |
|  | ART | **4 (1.2)** | **18 (6.6)** | **<.001** |

**Supplementary Table 2. Impact of Autopsy Decision and Cause of Death (COD) Findings Alone on Maternal Mental Health Outcomes 6-36 Months Post Stillbirth Delivery.**

| **Outcome** | **Secondary: Autopsy Decision** | | | | **Secondary: COD findings** | | |
| --- | --- | --- | --- | --- | --- | --- | --- |
|  | **No**  **Autopsy**  N=39 | **Partial**  **Autopsy**  N=52 | **Full**  **Autopsy**  N=181 | **p** | **Known**  **COD**  N=157 | **Unknown**  **COD**  N=115 | **p** |
| **Number of ‘Poor’ maternal mental health outcomes** | | | | | | | |
| 0 | 20 (51.3) | 24 (46.2) | 80 (44.2) | 0.391 | 72 (45.9) | 52 (45.2) | 0.726 |
| 1 or more | 19 (48.7) | 28 (53.8) | 101 (55.8) | 0.732 | 85 (54.1) | 63 (54.8) | >0.99 |
| 1 | 11 (28.2) | 15 (28.8) | 53 (29.3) |  | 44 (28.0) | 35 (30.4) |  |
| 2 | 2 (5.1) | 8 (15.4) | 30 (16.6) |  | 26 (16.6) | 14 (12.2) |  |
| 3 | 5 (12.8) | 2 (3.8) | 11 (6.1) |  | 9 (5.7) | 9 (7.8) |  |
| 4 | 1 (2.6) | 2 (3.8) | 7 (3.9) |  | 6 (3.8) | 4 (3.5) |  |
| 5 | 0 (0.0) | 1 (1.9) | 0 (0.0) |  | 0 (0.0) | 1 (0.9) |  |
| **Number of women with ‘poor’ scores on individual scales or subscales** | | | | | | | |
| Edinburgh Depression Scale (EPDS) ≥11 | 11 (28.9) | 17 (32.7) | 44 (24.3) | 0.449 | 37 (23.6) | 35 (30.7) | 0.211 |
| Perinatal Grief - Any Subscale score> 33 | 6 (15.4) | 9 (17.3) | 36 (19.9) | 0.779 | 33 (21.0) | 18 (15.7) | 0.276 |
| Perinatal Grief - Active Grief subscale >33 | 6 (15.8) | 9 (17.3) | 34 (18.8) | 0.917 | 32 (20.4) | 17 (14.9) | 0.267 |
| Perinatal Grief - Difficulty Coping subscale >33 | 6 (15.8) | 9 (17.3) | 34 (18.8) | 0.917 | 32 (20.4) | 17 (14.9) | 0.267 |
| Perinatal Grief Despair subscale >33 | 0 (0.0) | 1 (1.9) | 3 (1.7) | 0.854 | 3 (1.9) | 1 (0.9) | 0.641 |
| Impact of Events Scale (IES)> 44 | 10 (26.3) | 13 (25.0) | 44 (24.3) | 0.977 | 39 (24.8) | 28 (24.6) | >0.99 |
| Posttraumatic Growth Inventory (PGTI) <42 | 2 (5.3) | 4 (7.7) | 23 (12.7) | 0.292 | 19 (12.1) | 10 (8.8) | 0.431 |
| Increased smoking / drinking | 5 (13.5) | 7 (13.5) | 27 (15.4) | 0.903 | 19 (12.5) | 20 (17.9) | 0.292 |
| **Scores on individual scales or subscales** | | | | | | | |
| Edinburgh Depression Scale (EPDS) | 7.8±5.6 | 8.4±5.0 | 7.0±5.2 | 0.188 | 7.1±5.0 | 7.7±5.5 | 0.288 |
| Perinatal Grief Scale - Active Grief subscale | 27.1±6.4 | 27.2±5.4 | 27.3±6.5 | 0.98 | 27.6±6.6 | 26.8±5.9 | 0.293 |
| Perinatal Grief Scale - Difficulty Coping subscale | 19.2±5.4 | 20.0±6.1 | 19.6±6.4 | 0.814 | 19.6±6.5 | 19.6±5.8 | 0.91 |
| Perinatal Grief Scale - Despair subscale | 19.1±5.8 | 18.4±5.6 | 19.2±6.2 | 0.694 | 19.2±6.1 | 18.9±6.0 | 0.779 |
| Impact of Events Scale (IES) | 34.6±16.0 | 30.1±18.0 | 30.6±18.6 | 0.419 | 31.0±18.3 | 31.2±18.0 | 0.938 |
| Posttraumatic Growth Inventory (PGTI) scale | 71.9±20.3 | 73.2±18.8 | 69.1±22.4 | 0.417 | 68.8±21.7 | 72.3±21.0 | 0.189 |
| Posttraumatic Growth |  |  |  |  |  |  |  |

**Figure A1. Maternal Mental Health Outcomes Over Time After Stillbirth**
